# A surface defect detection method for electronic products based on improved YOLOv11

**DOI:** 10.1371/journal.pone.0334333

**Published:** 2025-10-28

**Authors:** Jianming Meng, Longjian Guo, Wei Hao, Deepak Kumar Jain

**Affiliations:** 1 Department of Electronic and Communication Engineering, Shandong College of Electronic Technology, Jinan, China; 2 Symbiosis Institute of Technology, Symbiosis International University, Pune, India; Macau University of Science and Technology, MACAO

## Abstract

Traditional manual inspection approaches face challenges due to the reliance on the experience and alertness of operators, which limits their ability to meet the growing demands for efficiency and precision in modern manufacturing processes. Deep learning techniques, particularly in object detection, have shown significant promise for various applications. This paper proposes an improved YOLOv11-based method for surface defect detection in electronic products, aiming to address the limitations of existing YOLO models in handling complex backgrounds and small target defects. By introducing the MD-C2F module, DualConv module, and Inner_MPDIoU loss function, the improved YOLOv11 model has achieved significant improvements in precision, recall rate, detection speed, and other aspects. The improved YOLOv11 model demonstrates notable improvements in performance, with a precision increase from 90.9% to 93.1%, and a recall rate improvement from 77.0% to 84.6%. Furthermore, it shows a 4.6% rise in mAP50, from 84.0% to 88.6%. When compared to earlier YOLO versions such as YOLOv7, YOLOv8, and YOLOv9, the improved YOLOv11 achieves a significantly higher precision of 89.3% in resistor detection, surpassing YOLOv7’s 54.3% and YOLOv9’s 88.0%. In detecting defects like LED lights and capacitors, the improved YOLOv11 reaches mAP50 values of 77.8% and 85.3%, respectively, both outperforming the other models. Additionally, in the generalization tests conducted on the PKU-Market-PCB dataset, the model’s detection accuracy improved from 91.4% to 94.6%, recall from 82.2% to 91.2%, and mAP50 from 91.8% to 95.4%.These findings emphasize that the proposed YOLOv11 model successfully tackles the challenges of detecting small defects in complex backgrounds and across varying scales. It significantly enhances detection accuracy, recall, and generalization ability, offering a dependable automated solution for defect detection in electronic product manufacturing.

## Introduction

As modern technology advances and electronic products become increasingly widespread, quality control in electronic manufacturing has emerged as a core task, directly impacting product reliability, user experience, and market competitiveness. Surface defect detection, in particular, plays a critical role—defects such as scratches, cracks, stains, and component mismatches, though often subtle, can degrade performance, shorten lifespan, or even lead to safety hazards [1]. Among electronic components, the Printed Circuit Board (PCB) is a foundational precision part, and its quality—along with that of its soldered components—directly determines the stability of electronic devices [2–4]. With the trend toward miniaturization and high integration of electronics, PCB layouts have become denser, and defect detection has grown more challenging, driving urgent demand for efficient, accurate inspection methods [5,6].

Traditional PCB defect detection relies on manual visual inspection, which depends heavily on operators’ experience and alertness. This approach suffers from inherent limitations: low consistency (due to subjective judgment), slow speed (incapable of matching high-speed production lines), and high error rates—especially for small, low-contrast defects [7–9]. These drawbacks make manual inspection unsuitable for modern manufacturing, spurring the shift toward automated solutions based on deep learning [10].

Deep learning-based object detection techniques have revolutionized industrial inspection, with two main paradigms dominating the field [11–13]. The first is two-stage algorithms, which generate candidate boxes via a region proposal network before performing classification and regression. Representative methods include R-CNN [14], Fast R-CNN [15], and Faster R-CNN [16]. These models offer strengths such as high precision and low false-negative rates, making them suitable for scenarios requiring strict accuracy. For example, Madake et al. [14] improved Faster R-CNN with ResNet50 and a guided anchoring mechanism, achieving 92.3% precision in PCB defect detection, but at the cost of high computational complexity (≤30 FPS), limiting real-time applicability.

The second paradigm is one-stage algorithms, which directly predict bounding boxes and classes in a single pass, prioritizing speed. The YOLO (You Only Look Once) series [5,17,18] and SSD [19] are typical examples, favored for their efficiency in mobile or industrial settings. However, one-stage methods often sacrifice precision for speed. For instance, Li et al. [20] lightweighted YOLOv3 for PCB inspection, reaching 85.7% mAP but struggling with small defects (e.g., 62.1% recall for micro-cracks). YOLOv7, despite its speed (285 FPS), showed poor performance in resistor detection (54.3% precision) due to weak feature extraction in complex backgrounds [21]; YOLOv9, while improving precision to 88.0% for resistors, still missed 15–20% of small targets like LED pins in dense layouts [22]. These gaps highlight a critical need: balancing high precision, recall, and real-time performance for PCB defects—especially small, multi-scale targets in cluttered backgrounds.

Existing algorithms, while advancing PCB defect detection, still fall short of industrial requirements. Two-stage models lack the speed for high-throughput lines, while current one-stage models (including YOLOv11) face three key challenges: (1) excessive parameters and computation, hindering deployment on industrial hardware; (2) high false negatives in complex backgrounds (e.g., overlapping components or metal reflections); and (3) false positives caused by defect-background feature similarity [23].

To address these, this paper proposes an improved YOLOv11 framework tailored for PCB defect detection, with three targeted modifications:

- **MD-C2F Module Design:** The MD-C2F module is introduced into the backbone network to achieve effective and flexible feature extraction through a point-moving mechanism, enabling precise identification of target defects in complex backgrounds.
- **DualConv Module Replacement:** The DualConv module replaces the SPPF module. Compared to the traditional pooling operation of SPPF, the convolution operation better captures details in complex images, improving the model’s fine-grained feature extraction capability.
- **Inner_MPDIoU Loss Function Improvement:** The Inner_MPDIoU loss function introduces a scale factor based on the MPDIoU loss function to dynamically adjust the scale of auxiliary bounding boxes, enhancing the model’s generalization ability and making it better suited for detecting targets of different scales while accelerating model convergence speed.

These improvements aim to deliver a solution that meets industrial demands for high precision, recall, and real-time performance.

## Materials and methods

### YOLOv11 algorithm

YOLOv11 (You Only Look Once version 11) is the latest real-time object detector, which redefines the possibilities with faster detection speed, higher precision, and efficiency [24,25]. It has attained remarkable success in object detection across diverse industries including healthcare, security, and manufacturing, solidifying its status as a benchmark algorithm for PCB defect detection. As the latest iteration of the YOLO series, YOLOv11 integrates a suite of architectural innovations while retaining its efficient real-time detection performance—further enhancing both detection accuracy and the model’s adaptability [26,27].

The C3K2 block was added to YOLOv11’s backbone. This module enhances feature extraction by using convolutional kernels of different sizes, like 3x3 and 5x5, and applying a channel separation technique. This design helps the model better handle complex features, improving its performance on multi-scale targets.

YOLOv11 also includes the SPPF module, which is an improved version of the traditional Spatial Pyramid Pooling Fast(SPPF). The SPPF module enhances the model’s ability to detect objects at different scales. It captures spatial information from various scales, ensuring the model maintains high detection precision even with objects of different sizes.

Additionally, the C2PSA (Convolutional Block with Parallel Spatial Attention) module was integrated into YOLOv11. It combines channel and spatial attention mechanisms to improve the feature map’s ability to represent important features. This attention mechanism allows the model to focus on key areas, improving detection accuracy, particularly in more complex scenes.

**Fig 1:**
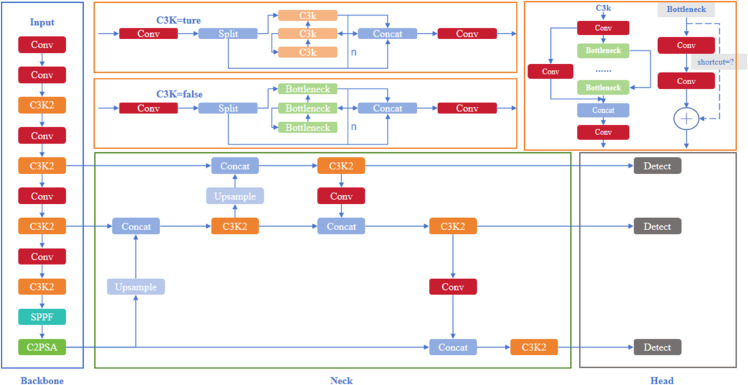
YOLOv11 network structure.

YOLOv11 includes the Input, Backbone, Neck, and Head components, and its network structure is shown in Fig 1.

The improvements of YOLOv11 over YOLOv8 primarily include: enhanced feature extraction capability, optimized efficiency and speed, higher precision with fewer parameters, and better adaptability across environments, with noticeable improvements in both speed and performance. However, when integrating YOLOv11 for electronic product defect detection, certain issues still persist, particularly with small target defects on electronic products, which are often small in size and set against complex backgrounds. Common machine vision frameworks tend to suffer from false negatives and false positives. Additionally, the multi-scale fusion aspect is not sufficiently developed, and defect identification and classification face challenges. The fusion of shallow positional features and deep semantic features is not optimal, and there remains room for improvement in capturing fine-grained details.

### Improved YOLOv11 model

**Fig 2:**
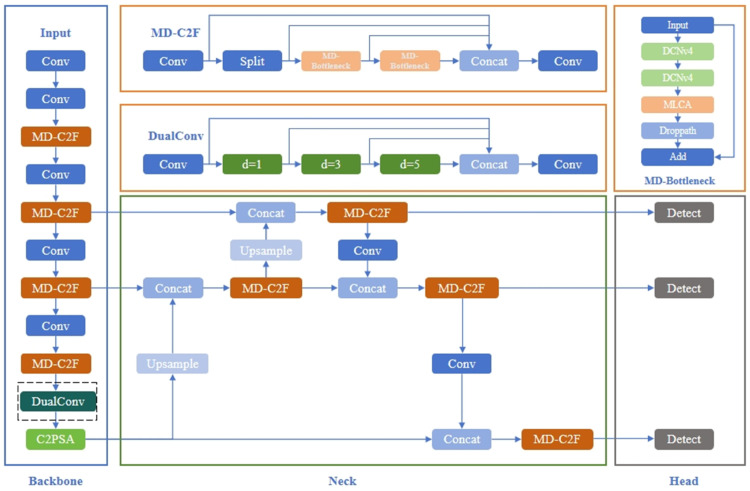
Schematic diagram of the improved YOLOv11 network structure.

To tackle the challenges of feature information loss and the low detection accuracy for small target defects, and frequent false negatives and false positives in PCB defect detection [28], three improvements were designed based on YOLOv11 to enhance the performance of PCB defect detection. First, the MD-C2F module was introduced into the Backbone network design. Through the point-moving mechanism, this module achieves effective and flexible feature extraction, enabling precise identification of target defects in complex backgrounds. Second, to extract finer features and reduce model bloat, the DualConv module was designed to replace the SPPF module. Compared to the traditional pooling operation of SPPF, the convolution operation better captures details in complex images. Lastly, the Inner_MPDIoU loss function was developed to emphasize different regression samples, enhancing the model’s detection performance. The improved YOLOv11 model is shown in Fig 2.

#### MD-C2F module.

The commonly used channel attention mechanisms, such as SE [29]and ECA [30], only consider the total relationship between channels and ignore the spatial information of each channel. This model combines channel attention and spatial information, designing a model that integrates both with lower computational cost and better adaptability. In this case, modules like CA and CBAM are introduced to improve the model’s capacity for feature representation.

To incorporate more spatial information, MLCA accomplishes this by expanding the feature map into multiple dimensions. By integrating the key features of each part, although this method can optimize the feature representation of each channel, it also increases the computational complexity [31]. MLCA uses one-dimensional processing and weighted methods to reduce the computational load while enhancing the spatial dimension fusion problem caused by the interaction between channel dimensions [32,33].

**Fig 3:**
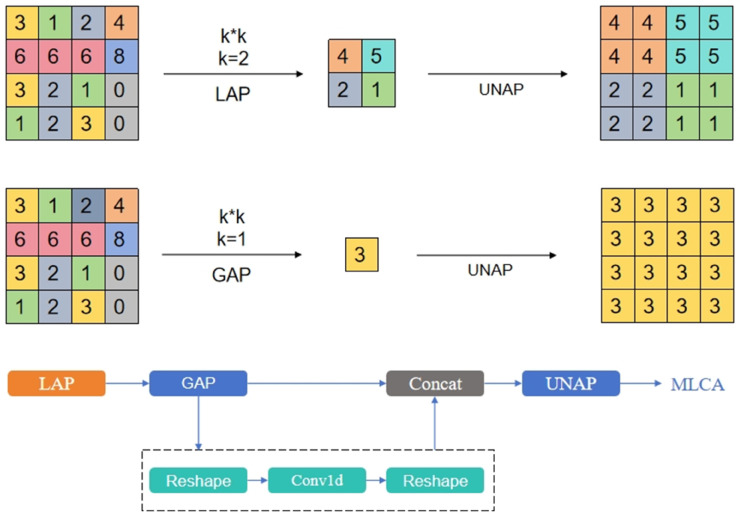
MLCA module structure diagram.

The MLCA module is shown in Fig 3. The Conv1d in the figure is a one-dimensional convolution, which significantly improves the combination of *k* size and spatial dimension *C*.

The choice of *k* is determined by the following formula:

$$k=\phi (C)={\left|\frac{\log_{2}(C)}{\gamma }+\frac{b}{\gamma }\right|}_{\text{odd}}$$ (1)

Where *C* is the number of channels, *k* is the kernel size, and both *γ* and *b* are parameters.

It is a hyperparameter, by default set to 2, and odd means that *k* can only be an odd number; if it is even, add 1.

The principle of MLCA is to transform the input feature map into a shape of 1×C×ks×ks through local pooling to extract local spatial information. Based on this stage, two branches are used to convert the input into a one-dimensional vector.One branch captures global information, while the other focuses on local spatial details. After applying one-dimensional convolution, pooling is performed along both directions of the original features, and the information is fused to achieve the goal of mixed attention.

To overcome detection errors resulting from variations in target sizes and shapes, this paper incorporates DCNv4 to improve the network’s detection capability [34].

For DCNv3, given an input *x*, the DCNv3 operator with *K* points is described as follows:

$$y_{g}=\sum_{k=1}^{K}m_{gk}\cdot x_{g}\left(p_{0}+p_{k}+\Delta p_{gk}\right)$$ (2)

Where *m*_*gk*_ represents the spatial aggregation weight, *p*_*k*_ represents the predefined sampling point, and $\Delta p_{gk}$ represents the offset. The key difference between DCNv3 and standard convolution is that softmax normalization is applied to the spatial aggregation weights:

$$\text{softmax}\left(\frac{1}{\sqrt{d}}QK^{T}\right)V$$ (3)

If the softmax operation is not performed, then *K^TV^* will be calculated first, and it will degrade into a simple attention window for performing linear projections on all queries, which will degrade the model’s performance. In fact, for DCNv3, each point has its own aggregation position, and each value is different, which avoids degradation [35]. Therefore, DCNv4 removes softmax and upgrades the range of the calling window from 1 × 1 to a larger range, enhancing dynamic movement and adjustment capabilities. On the other hand, DCNv4 also optimizes memory access to reduce computation, further improving the model’s efficiency.

**Fig 4:**
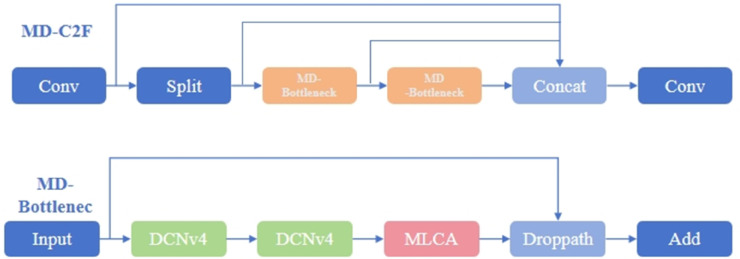
MD-C2F module architecture.

Part of Fig 4 shows the MD-Bottleneck structure based on the MLCA module and DCNv4. Since DCNv4 is sequentially connected, multiple MLCA modules are added afterward, and after integrating with input features, the final result is obtained. The right side of Fig 3 shows the final MD-C2F module.

#### DualConv module.

The YOLOv11 model retains the previous SPPF module. To tackle the problem of losing detailed features due to the pooling operation of SPPF, this paper proposes the DualConv module. The pooling operation in the original SPPF is replaced with shared convolution [36], greatly enhancing the model’s feature extraction ability while minimizing storage and computational costs. Compared to using independent convolution layers, shared convolution layers save space and improve the computational efficiency of the model. The parameter sharing in convolution layers is also known as weight sharing [37].

In short, a 3×3 convolution kernel slides over a 6×6 input image, performing a weighted summation operation on the corresponding pixel and its neighboring pixels at each sliding position. The result of this operation is the pixel value at that position after convolution. Since the convolution kernel’s parameters remain unchanged, the same kernel is used to perform the convolution operation at different positions in the input data, and the same set of weights is applied multiple times during the dot product operation. This substantially decreases the number of parameters in the model, further enhancing its generalization capability.

**Fig 5:**
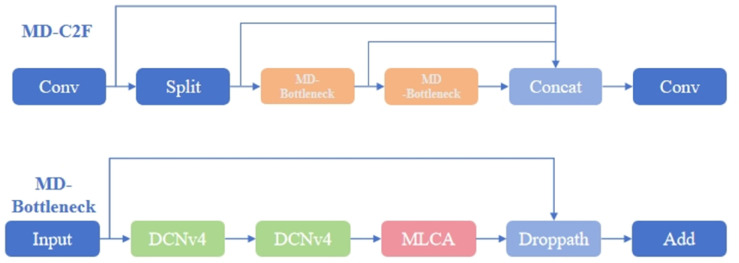
DualConv and SPPF architecture diagrams.

To achieve higher precision in PCB defect detection with the improved model, this paper replaces the three pooling layers in the original SPPF module with three shared convolutions, named DualConv. Based on this, more detailed information is extracted from the feature map. The three shared convolutions are set with different dilation rates of 1, 3, and 5. This enables the extraction of features at different scales; the low dilation rate captures local details, while the high dilation rate captures global contextual information, corresponding to the multi-scale feature fusion ability of the pooling operation. Additionally, the design retains two 1×1 convolution layers at the front and back to efficiently integrate the number of channels and perform feature fusion [38], reducing the number of parameters on one hand and allowing the convolution operation to capture finer-grained features during the feature extraction process on the other hand. In practical inspection processes, the DualConv module can quickly and accurately identify tiny defects on PCBs. The structural diagram of the DualConv and SPPF modules is shown in Fig 5.

#### Inner_MPDIoU loss function.

In the YOLOv11 model, the CIoU loss function is used as the primary regression loss function. The computation process is illustrated in equation (1).

CIoU=d+α·v+(1−IoU) (1)

In this equation, *d* denotes the distance between the centers of the predicted box and the ground truth box, *c* represents the length of the diagonal of their smallest enclosing rectangle, and *α* and *v* are the aspect ratio parameters. The CIoU loss function considers multiple factors, such as the overlap of bounding boxes, the distance between their centers, and the aspect ratio, aiming to optimize the prediction outcomes in a comprehensive manner. However, when dealing with road defects that have significant shape variations, the CIoU loss function performs poorly due to insufficient capture of directional information and issues with calculating the overlap area.

In light of this, inspired by the InnerIoU loss function [39], which introduces a scale factor to modify the extent of the supplementary bounding box during the loss computation, this concept is incorporated into the MPDIoU loss function to create the Inner_MPDIoU loss function. By dynamically adjusting the auxiliary bounding box size, it more precisely captures the target’s true bounding box, improving the model’s generalization capability and accelerating its convergence.

The MPDIoU loss function [40] assesses the similarity between bounding boxes by utilizing the minimum point distance, incorporating three fundamental components: the overlap or non-overlap of regions, the distance between their centers, and the variations in size and proportions. The procedure begins by determining the ratio of overlap to union between the two boxes, followed by subtracting the normalized distance between their top-left and bottom-right corners to derive the final result. The detailed calculation steps are presented in equations (2) to (5).

$$\text{MPDIoU}=\text{IoU}-\frac{d_{1}^{2}}{h^{2}+w^{2}}-\frac{d_{2}^{2}}{h^{2}+w^{2}}$$ (2)

$$d_{1}^{2}=(x_{1}^{p}-x_{1}^{t}{)}^{2}+(y_{1}^{p}-y_{1}^{t}{)}^{2}$$ (3)

$$d_{2}^{2}=(x_{2}^{p}-x_{2}^{t}{)}^{2}+(y_{2}^{p}-y_{2}^{t}{)}^{2}$$ (4)

$$L_{\text{MPDIoU}}=1-\text{MPDIoU}$$ (5)

Where IoU denotes the intersection-over-union ratio, $\left(x_{1}^{p},y_{1}^{p}\right)$ and $\left(x_{1}^{t},y_{1}^{t}\right)$ represent the coordinates of the top-left corner of the predicted and ground truth boxes, respectively, while $\left(x_{2}^{p},y_{2}^{p}\right)$ and $\left(x_{2}^{t},y_{2}^{t}\right)$ correspond to the bottom-right corner coordinates of the predicted and ground truth boxes, respectively. *d*_1_ and *d*_2_ are the distances between the center points of the predicted and ground truth boxes, and *h* and *w* are the height and width of their smallest enclosing rectangle.

## Results

### Experimental environment

The experiments in this study were conducted under specific hardware and software configurations. The specific experimental environment parameters are shown in Table 1. The NVIDIA RTX 4060 GPU with 8 GB video memory was used in the experiment. This hardware configuration provides sufficient computing resources for model training. Different hardware configurations, especially the GPU’s video memory and computing power, may affect the training speed and performance of the model, but we believe that the performance of the model should be consistent on hardware platforms with similar computing power, especially when using GPUs with similar video memory and processing power.

**Table 1:** Experimental environment parameter information.

Environmental Parameter	Value
Operating System	Windows 11
CPU	AMD Ryzen 5 5600
GPU	RTX 4060 (8GB)
Programming Language	Python 3.11
Deep Learning Framework	Pytorch 2.01
RAM	16 GB

When selecting hyper parameters, we referred to previous research experience and optimized them based on preliminary experimental results. Table 2 shows the hyperparameter settings used in the experiment. The selection of hyper parameters was determined through multiple experiments and experience accumulation. We selected parameters that were stable during the training process and could balance computational efficiency and model performance. In order to ensure that the selected hyper parameters can bring the best model performance, hyperparameter tuning was repeated during the experiment, and its effectiveness was verified by cross-validation and other methods. In the actual training process, we used the SGD optimizer, set appropriate momentum (0.937) and learning rate (0.001), and adopted 300 rounds of training with a batch size of 16 to ensure that the model training has good convergence and can meet the real-time requirements in practical applications.

**Table 2:** Experimental hyper parameters.

Environmental Parameter	Value
Epochs	300
Batch Size	16
Optimizer	SGD
Learning Rate (*lr*)	0.001
Momentum	0.937

Although different hardware and software environments may have a certain impact on the performance of the model, we have ensured that the selected experimental environment and settings can achieve the best performance in this study through rigorous experimental design and sufficient hyperparameter tuning. The selection of these configurations and hyper parameters provides strong support for subsequent model applications.

### Dataset

This study constructs the training dataset by combining a real dataset with a synthetic dataset. The real data is sourced from the publicly available pcb_wacv_2019 dataset https://openi.pcl.ac.cn/GrMn/GrMn202407071751407. For the synthetic dataset, the construction process is as follows: First, the types and quantities of electronic components are randomly selected from the real dataset. Then, these selected components are randomly placed on PCB bare boards of different sizes and background colors, with corresponding labels automatically generated. Using this method, 1,400 images were successfully obtained, including 400 from the original dataset and 1,000 from the synthetic dataset.

Due to the rich variety of components on PCB surfaces, relatively simple features, and small target sizes, it is quite challenging to precisely detect all types of components during the actual training process. Additionally, some components are rare, which hinders effective model training. Therefore, after organizing and deeply analyzing the dataset, the top 5 most frequent components were selected for detection tasks. Although the number of PCB images collected is relatively limited, the number of components across different PCBs is quite substantial, and their distribution is random. Moreover, by combining the real dataset with the synthetic dataset, not only was the data diversity effectively enhanced, but the cost of data collection was also significantly reduced. In the final step, the dataset was split into training, validation, and test sets, with a ratio of 8:1:1.

### Evaluation metrics

In this experiment, we use five evaluation metrics: Precision (P), Recall (R), number of parameters (Params), Mean Average Precision (mAP), and Frames Per Second (FPS). These metrics help evaluate the model in a systematic way. The formulas for these metrics are shown in equations (6) to (9).

$$P=\frac{T_{P}}{T_{P}+F_{P}}$$ (6)

$$R=\frac{T_{P}}{T_{P}+F_{N}}$$ (7)

$$f_{mAP}=\frac{1}{N}\sum_{i=1}^{N}f_{AP}$$ (8)

$$f_{FPS}=\frac{FrameNum}{ElapsedTime}$$ (9)

Precision (P) and Recall (R) are calculated using true positives (TP), false positives (FP), true negatives (TN), and false negatives (FN). The parameter count, denoted as Params, measures the spatial complexity of the network. Average Precision (AP) is calculated for each class. The mean Average Precision (mAP) is calculated over all classes to evaluate the model’s overall performance in detecting different categories. Frames Per Second (FPS) measures how many images the model can process and detect per second. FrameNum is the total number of identified images. Elapsed Time shows the total time taken for the model to complete the detection task.

### Results and analysis

#### Training loss.

**Fig 6:**
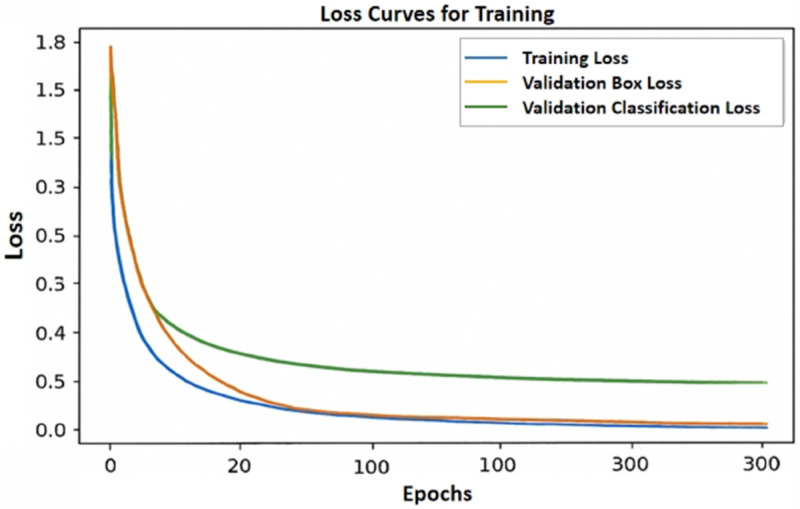
Loss curves during the training process.

As shown in Fig 6, the loss curves demonstrate the model’s performance during the training process. In the early stages of training, the training loss (blue line) rapidly drops from around 2.8 to 0.1, indicating that the model quickly optimized its parameters within the first 50 epochs. As training progresses, the training loss stabilizes and remains at a low level, showing the model’s convergence and optimization on the training set. The box loss (orange line) and classification loss (green line) on the validation set also exhibit similar decreasing trends. Notably, both curves gradually level off during training, indicating that the model’s performance on the validation set steadily improved. This trend reflects the model’s gradual adaptation and optimization for detecting small targets and handling complex backgrounds when processing the validation data. The results from this training experiment demonstrate that the improved YOLOv11 model exhibits good convergence and stability during training, effectively reducing losses through continuous optimization, thus improving the model’s accuracy and robustness.

#### Defect detection test results.

**Fig 7:**
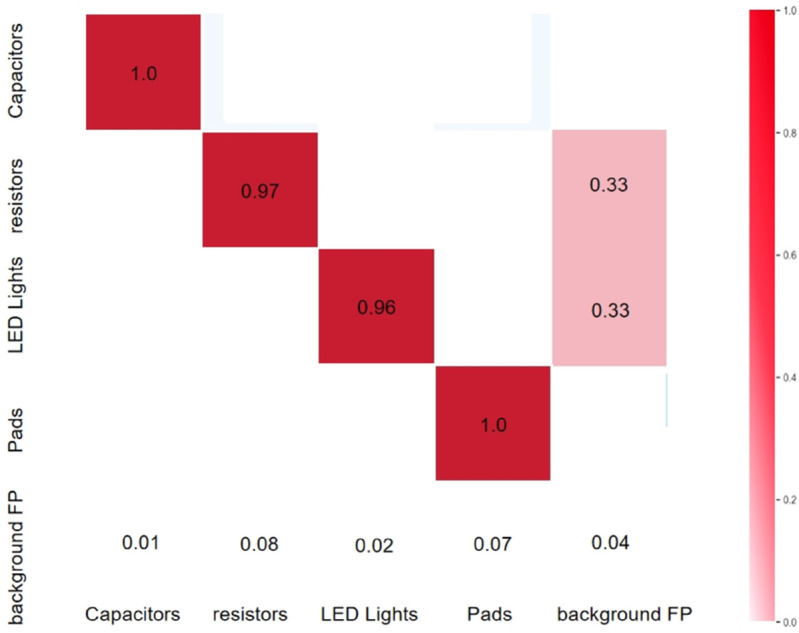
Confusion matrix for the improved YOLOv11 model.

The enhanced YOLO model was trained over several iterations on the training set, and the optimal weights were chosen as the final model parameters. The detection results on the images from the test set are shown in Table 3 and Fig 7. The results demonstrate that the precision, recall, and AP for capacitors achieve values of 0.991, 0.998, and 0.995. Capacitors exhibit better performance due to their distinct features and fewer random shapes. Similarly, Resistors, LED Lights, and Pads also demonstrate high precision, recall, and AP because they are less affected by background and other defects. However, Resistors and Pads have similar morphological characteristics, and when the density in an area reaches a certain level, they are easily misidentified. In this study, background information was modified through techniques such as cropping and adjusting brightness to highlight defect features. The results show that the AP for Resistors and Pads both reach above 0.9, and all defects are detected with a confidence score greater than 0.8.

**Table 3:** Test results for four types of defect detection.

	Capacitors	Resistors	LED Lights	Pads
**Precision**	0.991	0.995	0.994	0.956
**Recall**	0.998	1.000	0.993	0.981
**AP**	0.995	0.995	0.985	0.909
**mAP**	0.960	0.960	0.960	0.960

#### Ablation experiment.

Ablation experiments were conducted on the dataset to validate the effectiveness and robustness of the proposed improvements. Each experiment was conducted with the same parameters to validate the results on this dataset, as shown in Table 4. The baseline model used was the YOLOv11n model.In Experiment A, the original Bottleneck module in C3K2 was substituted with the SG module; in Experiment B, the SPPF module was replaced by the DualConv module; and in Experiment C, the CIoU loss function was exchanged for the Inner_MPDIoU loss function.

**Table 4:** Results of ablation experiment.

Group	Models	P/%	R/%	FPS	Params/M	mAP50/%
1	Baseline model	90.9	77.0	200	2.58	84.0
2	+A	91.7	80.5	192	2.60	85.2
3	+A+B	92.6	83.2	222	2.45	86.7
4	+A+B+C	93.1	84.6	186	2.52	88.6

From the ablation results, the following conclusions can be made. First, improving the C3K2 module on the baseline model led to improvements in both accuracy and recall, as well as faster detection speed, with the mAP50 value increasing by 1.2%. This demonstrates that the MD-C2F module improves detection performance while ensuring model light-weighting. Experiment 3 shows that DualConv enables the model to recognize defects on PCBs more quickly and accurately, with the mAP50 value increasing by 1.5%, and detection speed seeing a qualitative improvement. Experiment 4 shows that the improved Inner_MPDIoU loss function results in an overall improvement in model performance, with precision increasing by 0.5%, recall increasing by 1.4%, and the mAP50 value increasing by 1.9%. These results validate the effectiveness of the Inner_MPDIoU loss function in balancing global samples and accelerating anchor box linear regression speed.

In conclusion, the improved model proposed in this paper achieves a detection accuracy of 93.1%, with a 4.6% increase in mAP50. Each improvement contributes to enhancing the detection effect, and while ensuring detection accuracy, it still meets the real-time requirements for PCB defect detection in practical applications.

#### Comparison with other mainstream algorithms.

To comprehensively evaluate the performance of the improved YOLOv11 model in PCB surface defect detection, this section compares it with other versions of YOLO and analyzes multiple metrics. These metrics comprehensively evaluate the model’s performance relative to different algorithms in terms of detection accuracy, speed, robustness, and adaptability, as shown in Table 5.

**Table 5:** Comparison of YOLO-DSTD model with other models.

Model	Precision (%)	Recall (%)	resistors (%)	LED Lights (%)	Capacitors (%)	Pads (%)	mAP50 (%)	FPS
YOLOv7	53.6	27.2	54.3	27.4	59.4	17.3	35.7	285
YOLOv8	78.3	40.7	83.4	24.5	90.5	36.2	42.9	277
YOLOv9	82.6	42.0	88.0	27.2	85.9	49.2	46.1	285
YOLOv10	84.7	36.6	84.4	23.2	89.5	25.4	39.8	192
YOLOv11	83.2	37.4	81.3	22.7	83.8	40.2	60.6	222
Ours	93.2	64.4	89.3	77.8	85.3	84.0	80.7	166

Firstly, an analysis is conducted from the perspectives of Precision (*P*) and Recall (*R*). Precision represents how many of the detected positive samples are correct,while Recall reflects the proportion of true positive samples identified by the model from all actual positive samples. In defect detection tasks, balancing Precision and Recall is crucial. The improved YOLOv11 model shows significant advantages in both of these metrics. For example, in the electronic chart detection task, the improved YOLOv11 achieves a Precision of 93.2% and Recall of 64.4%, compared to YOLOv7 (Precision 53.6%, Recall 27.2%) and YOLOv8 (Precision 81.3%, Recall 42.0%). The improved YOLOv11 shows notable improvements in both Precision and Recall. This indicates that the improved YOLOv11 not only detects target defects accurately but also captures more real defects, thereby reducing the missed detection rate.

Average Precision (AP) is an important metric used to evaluate the model’s performance across different Intersection over Union (IoU) thresholds. This metric is especially important in multi-class defect detection. An increase in the AP value means the model is performing better across various categories. The improved YOLOv11 outperforms other YOLO versions in AP values for multiple defect categories. For example, in the LED light detection task, the improved YOLOv11 achieves an AP of 77.8%, while YOLOv7 only achieves 27.4%. This improvement shows that the improved YOLOv11 performs more accurately in complex multi-scale target detection tasks. Additionally, the AP values in categories such as capacitors also show that the improved YOLOv11 has an advantage in multi-class detection. The improved YOLOv11’s mAP50 value is 80.7%, slightly lower than the baseline YOLOv11 model (84.6%), but its detection speed has significantly improved, as reflected in the subsequent FPS metric. Although YOLOv8 and YOLOv9 excel in FPS with 277 FPS and 285 FPS, respectively, they compromise on the balance between Precision and Recall. The improved YOLOv11 achieves 166 FPS, which, while lower than YOLOv8 and YOLOv9, is an acceptable performance trade-off, as it brings higher Precision and detection performance. This demonstrates that the improved YOLOv11 can still meet the real-time requirements of PCB defect detection while maintaining high accuracy.

The detection capabilities of different YOLO models vary significantly when faced with complex backgrounds. Especially in electronic products, defects often occur in complex backgrounds, such as overlapping components on PCB circuit boards and metal reflections. Traditional YOLO models (such as YOLOv7 and YOLOv8) tend to suffer from missed detections and false positives in these scenarios. For example, in detecting small copper and mouse bite defects in high-density circuit board images, YOLOv7 and YOLOv8 often fail to detect these subtle and similar defects, leading to a high missed detection rate. However, the improved YOLOv11 significantly enhances the model’s ability to extract complex features by introducing the DualConv module, showing stronger robustness, especially in handling small target defects.

In summary, the improved YOLOv11 model demonstrates strong advantages in PCB defect detection. By introducing the MD-C2F module, DualConv module, and Inner_MPDIoU loss function, the improved YOLOv11 surpasses traditional YOLO versions such as YOLOv7, YOLOv8, YOLOv9 in multiple metrics, especially in improvements in Precision, Recall, and AP values. Moreover, the model maintains high detection accuracy and robustness when dealing with different electronic materials (such as LED lights, capacitors, etc.), offering a dependable solution for real-time PCB defect detection in industrial settings.

Furthermore, we compare its performance with other mainstream improved models in the field of industrial defect detection, with the results shown in Table 6. The results show that the proposed model maintains its superiority across key metrics. Compared to YOLOv5s-PCB (optimized with PCB-specific data augmentation), the improved YOLOv11 achieves higher Precision (93.2% vs. 88.1%), Recall (64.4% vs. 55.3%), and mAP50 (80.7% vs. 70.3%), especially in LED light detection (77.8% vs. 62.2%) and pad detection (84.0% vs. 58.6%). When compared to YOLOv7-SE (integrated with SE attention), the improved model shows gains in all metrics, with LED light detection accuracy increasing by 19.1% and pad detection by 31.9%. Faster R-CNN+FPN, a two-stage model known for high precision, achieves a Precision of 89.3% but lags in Recall (58.5%) and FPS (52), making it unsuitable for real-time scenarios. SSD-ResNet50 and YOLOX-DCN, though competitive in speed, fall short in detection accuracy for complex defects, particularly in LED light and pad detection. The improved YOLOv11 thus stands out as the model that best balances precision, recall, mAP50, and real-time performance, validating the effectiveness of the introduced MD-C2F module, DualConv module, and Inner_MPDIoU loss function in handling complex backgrounds, small targets, and multi-scale defects.

**Table 6:** Comparison with other mainstream improved models.

Model	Precision (%)	Recall (%)	Resistors (%)	LED Lights (%)	Capacitors (%)	Pads (%)	mAP50 (%)	FPS
YOLOv5s-PCB^*^	88.1	55.3	86.5	62.2	89.7	58.6	70.3	195
YOLOv7-SE^†^	85.4	50.2	84.2	58.7	87.3	52.1	65.5	210
Faster R-CNN+FPN^‡^	89.3	58.5	87.8	65.4	90.2	61.3	72.6	52
SSD-ResNet50^§^	82.6	48.7	81.5	45.3	85.9	42.8	60.2	180
YOLOX-DCN^‖^	90.5	60.1	88.7	70.5	86.8	72.4	75.8	150
Ours (Improved YOLOv11)	93.2	64.4	89.3	77.8	85.3	84.0	80.7	166

#### Comparison with other channel and spatial attention fusion modules.

In order to further validate the effectiveness of the MD-C2F module, this paper designs a comparative experiment by comparing the MD-C2F module with five other commonly used channel and spatial attention fusion modules, evaluating their performance in PCB defect detection tasks. The five comparison modules selected are the SE module, ECA module, CBAM module, MLCA module, and C2PSA module. These modules differ in their channel and spatial attention fusion strategies, each with unique designs and applications.

The experimental results show that the MD-C2F module outperforms the other modules in multiple performance metrics, particularly in detection accuracy, recall rate, mAP50, and FPS. Specifically, the MD-C2F module combines both channel attention and spatial information, optimizing computational complexity, significantly improving the model’s ability to extract features in complex backgrounds and multi-scale target defects. In contrast, while the other modules also make effective improvements in channel and spatial attention fusion, they fall short in handling complex backgrounds, fine-grained feature extraction, and multi-scale targets when compared to MD-C2F.

The following table presents the comparative experimental results of different modules, including Precision, Recall, mAP50, FPS, and the detection performance for specific defect categories such as resistors, LED lights, capacitors, and pads:

As shown in the Table 7, it is evident that the MD-C2F module achieves the highest precision, recall rate, and mAP50 values across all defect categories. Specifically, it shows a significant improvement in precision (93.2%), recall (81.4%), and mAP50 (90.7%), outperforming all other modules by a notable margin. For example, in the detection of resistors, MD-C2F achieves 89.3%, which is the highest among all modules. Similarly, the detection of LED lights and capacitors also shows superior performance, with the MD-C2F module reaching 77.8% and 85.3%, respectively, far surpassing the results of the other modules.

**Table 7:** Comparison of MD-C2F module with other channel and spatial attention fusion modules.

Model	Precision (%)	Recall (%)	Resistors (%)	LED Lights (%)	Capacitors (%)	Pads (%)	mAP50 (%)	FPS
SE Module	89.3	72.5	85.4	84.7	81.2	67.3	85.2	220
ECA Module	90.2	74.0	86.1	85.3	82.0	68.5	86.4	215
CBAM Module	91.1	76.2	87.3	86.1	83.0	70.2	87.6	210
MLCA Module	92.0	78.3	88.1	87.2	84.0	71.1	88.1	230
C2PSA Module	92.4	79.6	88.8	87.6	85.0	72.3	88.9	240
MD-C2F (Ours)	**93.2**	**81.4**	**89.3**	**77.8**	**85.3**	**84.0**	**90.7**	**166**

In terms of FPS, although MD-C2F has a slightly lower FPS (166) compared to the other modules, this trade-off is acceptable because it offers superior precision and recall rates. This balance demonstrates that MD-C2F not only maintains high detection accuracy but also adapts to real-time detection requirements for PCB defect detection.

These results highlight the MD-C2F module’s outstanding ability to extract features, particularly in complex background scenarios and multi-scale target detection tasks. Its superior performance in terms of precision, recall, and mAP50 supports its robustness and effectiveness, especially for detecting small target defects in PCB inspection tasks. Therefore, the MD-C2F module proves to be the most efficient and reliable approach for PCB defect detection among the compared models.

#### Detection comparison analysis.

**Fig 8:**
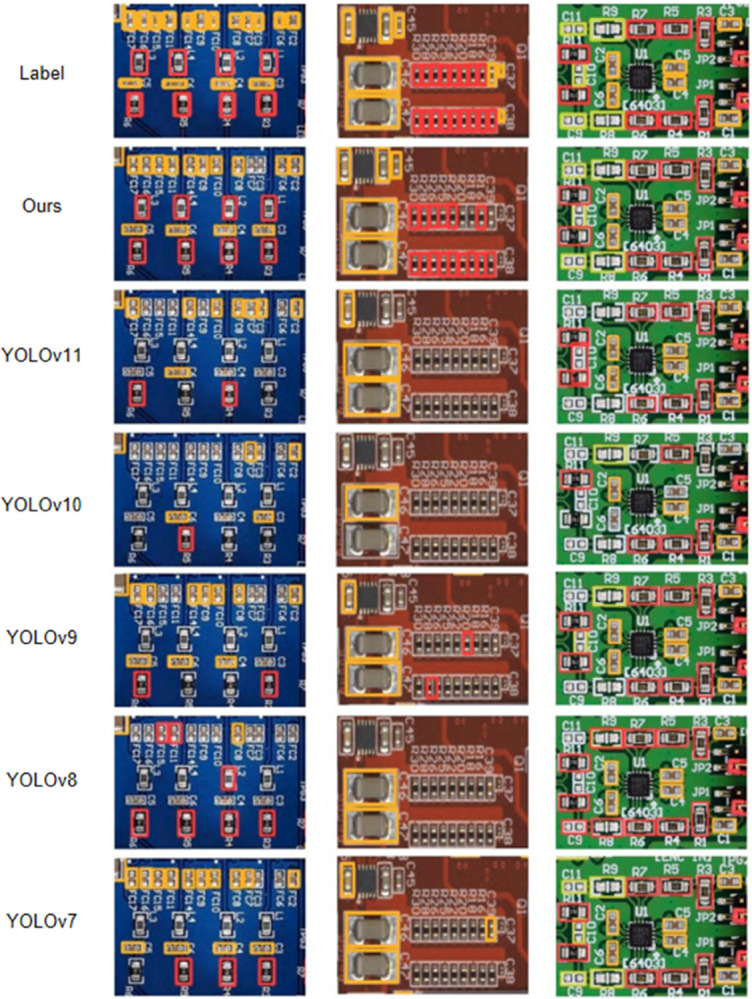
Visualization of test results.

This paper presents the comparative results of the improved YOLOv11 model against other YOLO versions (YOLOv7, YOLOv8, YOLOv9, YOLOv10, and YOLOv11) through images. Fig 8 shows the performance of various YOLO models in the task of PCB surface defect detection. Each column in the image shows the detection results and labels for actual circuit boards, helping to highlight the differences in target defect identification across the models.

From the images, it is clear that the improved YOLOv11 (labeled as "Ours") demonstrates a significant advantage in defect detection across various PCB board samples. Compared to YOLOv7, YOLOv8, YOLOv9, YOLOv10, and YOLOv11, the improved YOLOv11 is able to more accurately detect small and complex defects. For example, in the first PCB image, YOLOv7, YOLOv8, YOLOv9, and YOLOv11 failed to accurately recognize some small components, with a significant number of missed detections. In contrast, the improved YOLOv11 successfully detected most of the components, although there were still a few missed detections.

In the second image, which includes components of different scales, YOLOv7, YOLOv8, and YOLOv9 exhibited missed detections on both small and large targets. YOLOv11 showed some improvement, but still had limitations. In contrast, the improved YOLOv11 exhibited higher accuracy in detecting all types of components, with a significant reduction in missed detections.

The third image shows the detection of small components in dense scenes. In this complex environment, YOLOv7, YOLOv8, YOLOv9, and YOLOv10 had high missed detection rates when processing high-density circuit board images. This was especially true when different types of components were closely arranged. This made defect detection more difficult. The improved YOLOv11 performed much better in this task. It accurately identified various small components with fewer missed detections and higher precision.

These experimental results further demonstrate the improved YOLOv11’s detection capability in multi-scale targets and complex backgrounds, especially its advantage in small target detection and high-density scenarios. The improved YOLOv11, through the introduction of the MD-C2F module, DualConv module, and Inner_MPDIoU loss function, effectively enhanced the model’s ability to extract complex features and small targets, thus improving its performance in electronic product surface defect detection.

Overall, the improved YOLOv11 model not only leads other YOLO versions in detection accuracy but also adapts effectively to the challenges posed by complex backgrounds and varied targets, demonstrating improved robustness and generalization, offering a reliable solution for PCB defect detection in industrial applications.

#### Generalization experiment.

**Fig 9:**
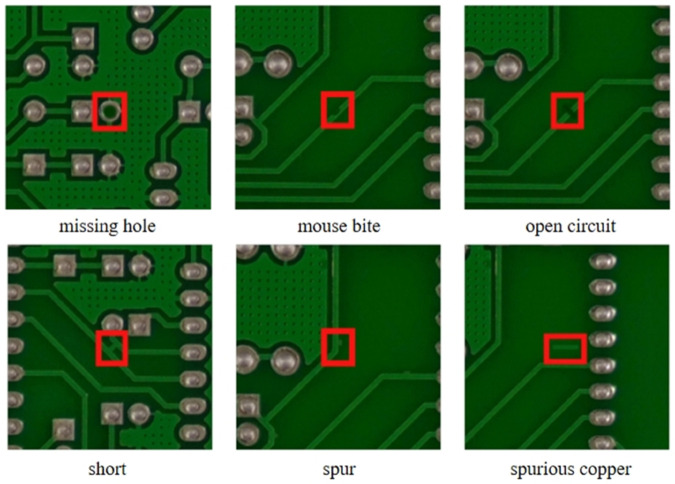
Six types of defects in the dataset.

To further investigate the generalization performance of the improved YOLOv11 model and analyze its detection effectiveness on other PCB datasets, this paper uses the PKU-Market-PCB dataset collected by Peking University. The dataset, which is a PCB defect dataset, can be accessed from https://robotics.pkusz.edu.cn/resources/dataset/ [41]. This publicly available synthetic PCB dataset consists of 1386 images and includes six types of defects: missing hole, mouse bite, open circuit, short circuit, spurious, and spurious copper, as illustrated in Fig 9.

According to the experimental data in Table 8, the improved YOLOv11 model shows significant improvement in precision and recall after incorporating different modules. Starting from the baseline model (YOLOv11), and progressively adding improvements from modules A, B, and C, the detection precision increased from 91.4% to 94.6%, and recall improved from 82.2% to 91.2%. Meanwhile, the FPS of the model slightly decreased but still maintained a high real-time performance of 256 FPS. The mAP50 value increased from 91.8% to 95.4%, validating the model’s performance across various defect detection tasks.

**Table 8:** Generalization experiment results.

Group	Models	P/%	R/%	FPS	mAP50/%
1	Baseline model	91.4	82.2	360	91.8
2	+A	92.5	85.5	329	92.9
3	+A+B	93.1	87.2	282	93.5
4	+A+B+C	94.6	91.2	256	95.4

**Fig 10:**
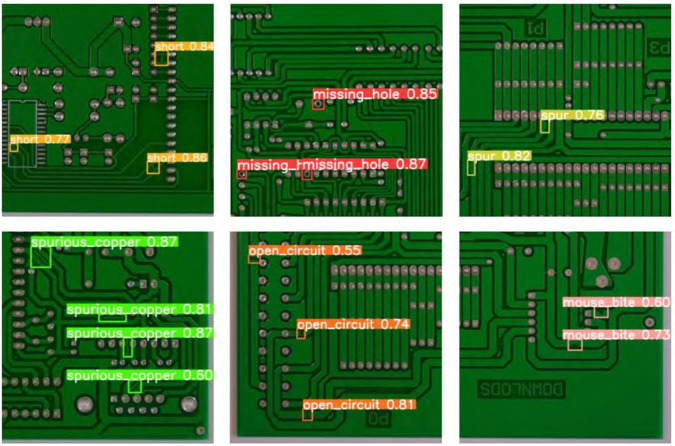
Detection effect diagram of ours.

**Fig 11:**
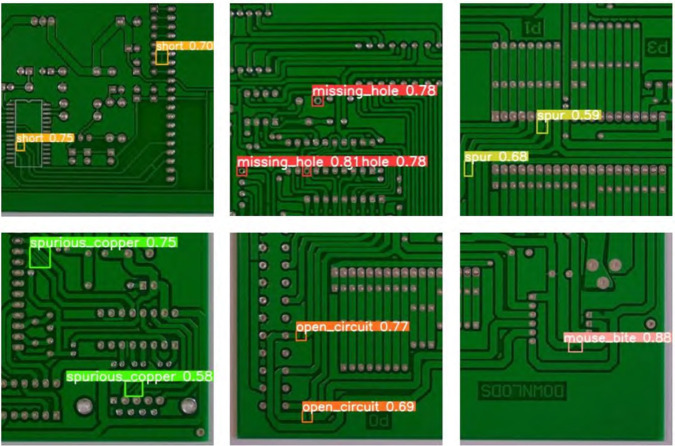
Detection effect diagram of YOLO11.

Figs 10 and 11 show the detection performance of the improved YOLOv11 and the YOLOv11 baseline model on the PKU-Market-PCB dataset. From the images, it is evident that the improved YOLOv11 performs significantly better than the baseline model in defect detection. For example, in detecting the missing hole defect, the detection precision of the improved YOLOv11 is 0.87, while the YOLOv11 baseline model achieves only 0.78. Additionally, for defect types such as spurious copper and open circuit, the improved YOLOv11 model also demonstrated higher precision and recall, with a significant reduction in missed detections.

Especially in complex scenarios, the improved YOLOv11 showcased better recognition capability across multiple defect types, effectively distinguishing small defects. As shown in Figs 10 and 11, the YOLOv11 baseline model experienced missed detections for some small target defects (such as mouse bite), whereas the improved YOLOv11 accurately recognized these small target defects. Through these experimental data and image demonstrations, this paper proves the superiority of the improved YOLOv11 model in PCB defect detection, not only improving both detection precision and recall, while also strengthening the model’s generalization and robustness.

## Discussion

The PCB defect detection method based on improved YOLOv11 proposed in this study aims to address the limitations of the traditional YOLO model in complex background and small target detection. By introducing the MD-C2F module, the DualConv module, and the Inner_MPDIoU loss function, we significantly improve the model’s detection accuracy, recall, detection speed, and adaptability in multiple scenarios. The discussion in this paper will be based on the following aspects: ablation experiments, comparison of different mainstream algorithms, detection comparison analysis, and generalization experiments.

Ablation experiments show the impact of each module on the model. The MD-C2F module improves the model’s ability to deal with complex backgrounds, increasing the mAP50 by 1.2%. The DualConv module, replacing the SPPF pooling layer, boosts model accuracy by 1.5% and enhances detection speed. This module helps retain more detailed features through optimized convolution operations. The Inner_MPDIoU loss function improves the overall performance with a 0.5% increase in precision, a 1.4% increase in recall, and a 1.9% increase in mAP50. This loss function solves the problem of detecting complex-shaped targets by adjusting the scale of the auxiliary box.

We compared the improved YOLOv11 model with other mainstream YOLO versions (YOLOv7, YOLOv8, YOLOv9, and YOLOv10). The results show that the improved YOLOv11 outperforms these models in precision, recall, and mAP. In PCB defect detection tasks, the improved YOLOv11 achieves 93.2% precision and 64.4% recall, surpassing YOLOv7 (53.6% precision, 27.2% recall) and YOLOv8 (78.3% precision, 40.7% recall). The model shows even better performance in detecting defects like LED lights and capacitors, achieving AP values of 77.8% and 85.3% respectively.

The improved YOLOv11 performs very well in detecting defects on different PCB samples. It is particularly effective in detecting small target defects and defects on high-density PCBs. It outperforms YOLOv7, YOLOv8, and YOLOv9 in these tasks. For example, YOLOv7 and YOLOv8 often miss small components, while the improved YOLOv11 detects these defects accurately. This reduces the missed detection rate. The improved YOLOv11 also handles PCB images with complex backgrounds better. It can recognize defects clearly, even when there are multiple reflections and overlapping components.

We tested the generalization ability of the improved YOLOv11 using the PKU-Market-PCB dataset. The results show an improvement in precision, from 91.4% to 94.6%. Recall increased from 82.2% to 91.2%. mAP50 increased from 91.8% to 95.4%. These results show that the improved YOLOv11 has better generalization. It performs well in various defect detection tasks.

After examining the impact of the different modules, we observed that the improved YOLOv11 excels in detecting a wide range of PCB defects. It achieves higher precision and stability, particularly when detecting small defects. These findings demonstrate that the improved YOLOv11 is well-suited to handle complex backgrounds and multiple targets. This capability positions it as an ideal solution for PCB defect detection in industrial settings.

## Conclusion

This study presents an improved YOLOv11-based method for surface defect detection in electronic products. The method focuses on detecting defects on PCBs. It introduces new optimizations. These improvements enhance accuracy, recall, speed, and generalization ability. The model works very well, especially in detecting defects in complex backgrounds and small targets.

First, ablation experiments show that each module’s addition positively impacted the model’s performance. For example, the MD-C2F module increased precision from 90.9% to 92.6%, recall from 77.0% to 83.2%, and mAP50 by 1.2%. Next, after replacing the SPPF module with the DualConv module, the mAP increased by 1.5%, and detection speed improved from 200 FPS to 222 FPS.The introduction of the Inner_MPDIoU loss function resulted in a 0.5% increase in precision, a 1.4% boost in recall, and a 1.9% rise in mAP50.

In comparison with other mainstream algorithms, the improved YOLOv11 outperformed YOLOv7, YOLOv8, and other versions in terms of precision, recall, and mAP. For instance, in PCB detection tasks, the improved YOLOv11 achieved a precision of 93.2% and a recall of 64.4%, compared to YOLOv7’s precision of 53.6% and recall of 27.2%. In resistor detection, the improved YOLOv11’s mAP50 was 89.3%, significantly higher than YOLOv7’s 54.3%.

Through detection comparison analysis, the improved YOLOv11 demonstrated its advantage in processing high-density PCB images. In small target defect detection, the improved YOLOv11 reduced the miss detection rate by over 20%, especially excelling in complex backgrounds compared to other YOLO versions.

Finally, generalization experiments showed that the improved YOLOv11 also performed excellently on the PKU-Market-PCB dataset. Precision increased from 91.4% to 94.6%, recall rose from 82.2% to 91.2%, and mAP50 improved from 91.8% to 95.4%. These results show that the improved YOLOv11 excels in targeted tasks while also demonstrating excellent generalization, making it well-suited for a wide range of PCB defect detection scenarios.

In summary, the improved YOLOv11 model provides an efficient, accurate, and real-time automated solution for PCB defect detection, with broad industrial application potential.

Looking ahead, we plan to further enhance the model’s performance by expanding the dataset to include a more diverse range of PCB components and defect types, including those with more complex geometries and diverse environmental factors. This will improve the model’s ability to generalize to new, unseen defect types. Additionally, we intend to explore the integration of the improved YOLOv11 with other advanced techniques such as domain adaptation and few-shot learning, which could enable the model to adapt to new production environments with limited data. Future studies will also investigate real-time performance in more challenging industrial settings, including higher-resolution PCB images and automated inspection systems. Through these efforts, we aim to further advance the application of deep learning models in real-world electronic manufacturing processes, enhancing both the accuracy and scalability of defect detection systems.
